# Humble Leadership Affects Organizational Citizenship Behavior: The Sequential Mediating Effect of Strengths Use and Job Crafting

**DOI:** 10.3389/fpsyg.2020.00065

**Published:** 2020-02-13

**Authors:** He Ding, Enhai Yu, Xixi Chu, Yanbin Li, Kashif Amin

**Affiliations:** ^1^School of Economics and Management, North China Electric Power University, Beijing, China; ^2^School of Labor and Human Resources, Renmin University of China, Beijing, China

**Keywords:** humble leadership, strengths use, job crafting, organizational citizenship behavior, structural equation modeling

## Abstract

The purpose of the current study is to investigate the impact of humble leadership on organizational citizenship behavior (OCB) and the sequential mediation effect of strengths use and job crafting on the relationship. Data were collected at two points in time, spaced by a 2-week interval, with a sample of 260 employees working in a hospital in Beijing, China. Structural equation modeling was applied to test our predictions. The results indicated that humble leadership is positively related to OCB; the relationship between humble leadership and OCB was mediated not only by job crafting but also by strengths use and job crafting (sequential mediation). However, the indirect effect of humble leadership on OCB through the mechanism of strengths use was not statistically significant. This study advances the theory and research on the leadership–OCB relationship.

## Introduction

More than five decades ago, Katz (1964) suggested that a class of discretionary and spontaneous behaviors beyond explicit role requirements is of great importance for organizational effectiveness. Smith et al. (1983) named such behaviors as “organizational citizenship behavior” (OCB). Subsequently, OCB was defined as “individual behavior that is discretionary, not directly or explicitly recognized by the formal reward system, and that in the aggregate promotes the effective functioning of the organization” (Organ, 1988, p. 4). OCB has received a great deal of attention among practitioners and scholars due to its positive effect on employees’ performance (e.g., Podsakoff and Mackenzie, 1997; Becton et al., 2017). Given the importance of OCB for organizational effectiveness (Podsakoff and Mackenzie, 1997), numerous researchers have directed their research interests toward the antecedents to OCB.

To date, extant research has identified many determinants of OCB such as perception of organizational politics (Khan et al., 2019), emotional intelligence (Lim et al., 2018), perceived organizational support (Dai et al., 2018), psychological contract fulfillment (Ahmad and Zafar, 2018), and ethical leadership (Yang and Wei, 2017; Mostafa, 2018). However, little is known about whether humble leadership can affect OCB, and the potential mechanisms accounting for this relationship is underdeveloped. We thus aim to redress these gaps by proposing a sequential mediation model.

Based on the existing literature on humble leadership and OCB, we first expect that humble leadership is related to higher levels of OCB, because subordinates who follow humble leadership tend to experience high levels of self-efficacy (Wang et al., 2018), with meaning in work and self-determination (Chen et al., 2018) as significant predictors of OCB (Schlechter and Maharaj, 2007; Yan and Chen, 2013; Kao, 2017). The second purpose of this study is to examine the mediating role of strengths use in the humble leadership–OCB linkage. Owens and Hekman (2012) have suggested that humble leadership is characterized by appreciating subordinates’ strengths, which can motivate subordinates to leverage their strengths at work (Govindji and Linley, 2007), ultimately resulting in an enhanced OCB (Van Woerkom and Meyers, 2015). In addition, we also test whether job crafting acts as a crucial mediator between humble leadership and OCB. The reason for this aim is that job crafting, as a proactive behavior which employees take to improve person–job fit (Wrzesniewski and Dutton, 2001; Tims et al., 2012), might benefit more from humble leadership (Chen et al., 2018) and can lead to increased OCB (Bavik et al., 2017). Importantly, since strengths use is associated with job crafting (Plomp et al., 2016), we also examine whether humble leadership will affect subordinates’ OCB by strengths use and job crafting (sequential mediation).

With this study, we attempt to make two aspects of theoretical contributions. First, although leadership as a critical contextual factor such as transformational leadership (Podsakoff et al., 1990) and servant leadership (Walumbwa et al., 2010) has been revealed to relate to high levels of OCB, no prior study has investigated the relationship between humble leadership and OCB. Thus, this paper enriches the existing research on the leadership–OCB relationship by testing the association of humble leadership with OCB. Second, previous research concerning the leadership–OCB linkage has identified the mediating mechanisms between humble leadership and OCB from the perspectives of attitude or motivation (e.g., Nasra and Heilbrunn, 2016; Newman et al., 2017); very little research focused on positive behaviors as mediating mechanisms linking humble leadership to OCB. By investigating the mediating (and sequentially mediating) roles of strengths use and job crafting in the humble leadership–OCB relationship, this study contributes to unlocking the “black box” of the relationship between humble leadership and OCB and extends our understanding of the underlying mechanisms between humble leadership and OCB.

### Theory and Hypothesis Development

#### Humble Leadership and OCB

Humility leadership representing social interpersonal characteristics (Owens and Hekman, 2012) consists of three aspects: a willingness to view oneself accurately, an appreciation of others’ strengths and contribution, and teachability (Owens et al., 2013). In recent years, the construct of humble leadership has received remarkable scholarly attention (Owens et al., 2013). Extant literature has suggested that humble leadership makes subordinate growth and development legitimization (Owens and Hekman, 2012); promotes subordinate learning orientation, job satisfaction, work engagement, and retention (Owens et al., 2013); develops loyalty and commitment (Basford et al., 2014); elevates top management team integration and empowering climate (Ou et al., 2017); mitigates the negative impact of leader narcissism on positive follower outcomes (Owens et al., 2015); positively influences team performance through social contagion process (Owens and Hekman, 2016); and enhances subordinate creativity via psychological capital (Wang et al., 2018). Furthermore, humble leadership has been found to be related to team performance, and the relationship is mediated by team psychological capital and team task allocation effectiveness (serial mediation) (Rego et al., 2017a). In a similar vein, Rego et al. (2017b) also pointed out that humble leadership can increase team performance via enhanced collective humility and team psychological capital (sequential mediation). Liu et al. (2017) demonstrated that humble leadership can facilitate team innovation by shaping the team voice climate. Chen et al. (2018) identified a moderated mediation connection between humble leadership, identification with the leader, psychological empowerment, and employee proactive behavior. Although much research has stressed the importance of humble leadership to individuals and organizations, there is still a need for investigating the effect of humble leadership on a wider range of positive outcomes of individuals or organizations. Given that OCB plays a crucial role in facilitating organizational productivity, efficiency, and employees’ performance evaluations and promotions (Podsakoff et al., 2009), further research on the antecedents of OCB is of great significance. Thus, the present study aims to examine whether humble leadership can significantly improve employees’ OCB.

We speculate that humble leadership has a positive correlation with OCB. At least three reasons offer support for the prediction. First, Wang et al. (2018) demonstrated that when humble leaders appreciate subordinates’ strengths, even view themselves as students of their subordinates’ strengths (Owens and Hekman, 2012), subordinates’ perceptions of self-efficacy and self-worth will be enhanced. Given that self-efficacy has a close relation to OCB (Dussault, 2006; Kao, 2017), humble leadership might have a positive correlation with OCB. Second, the appreciation of subordinates’ contributions that humble leaders express can help subordinates recognize the importance of their contributions to organizations and, in turn, facilitates subordinates’ perception of meaning in their jobs (Chen et al., 2018). Considering the positive impact of the meaning of work on OCB (Schlechter and Maharaj, 2007), it is reasonable to anticipate that humble leadership is positively related to OCB. Third, humble leaders used to acknowledge their deficits and show themselves to be open to new ideas, which can provide subordinates with opportunities for self-determination (Chen et al., 2018). Such self-determination can lead to an increase in OCB (Yan and Chen, 2013). Accordingly, it is possible to postulate humble leadership to be linked with OCB. Based on the above arguments, the following hypothesis is derived:

**Hypothesis 1:** Humble leadership is positively related to OCB.

#### Humble Leadership and Strengths Use

Strengths use is defined as the proactive behaviors which employees take to deploy their strengths at work (Van Woerkom et al., 2016a). Researchers have done much valuable work to explore the driving forces of employee strengths use. For example, Govindji and Linley (2007) demonstrated that strengths knowledge is a significant predictor of strengths use. In other words, individuals who recognize their strengths are more likely to use their strengths. In a recent review of strengths use literature conducted by Bakker and van Woerkom (2018), it was indicated that stable personality traits might have a significant correlation with strengths use. For example, neuroticism and extraversion are significantly and negatively related to strengths use (Bakker et al., 2019); a structural equation modeling analysis has also found that independent self-construal could significantly and positively predict employees’ strengths use (Kong and Ho, 2016). Besides, a piece of research based on South African employees revealed that employees who gained more job resources (e.g., autonomy, information, and support for strengths use) were more apt to deploy their strengths in the workplace (Botha and Mostert, 2014). Consistent with the aforementioned results, Kong and Ho (2016) found that when employees perceived higher levels of autonomy support from leaders, they would have a stronger motivation to utilize strengths; Van Woerkom et al. (2016b) suggested that organizational support for strengths use (for instance, organizations help employees to identify their strengths) had a positive effect on strengths use indeed.

According to the above discussion, we know that both individual characteristics and situational circumstances have vital roles in elevating employee strengths use. It is important to note that leaders’ role may be considered as one of the more prominent influencing factors of strengths use (Kong and Ho, 2016). In the present research, we anticipate that humble leadership has a positive association with strengths use. First, since humble leadership legitimizes subordinates’ growth and development (Owens and Hekman, 2012) and previous literature demonstrates that strengths are the greatest room for subordinates’ growth and development (Buckingham and Clifton, 2001), subordinates who follow humble leadership will be more likely to take various behaviors to leverage their strengths at work to achieve a high level of growth and development. Second, humble leaders always show appreciation of subordinates’ strengths (Owens and Hekman, 2012), which conveys a piece of important information to subordinates that leaders encourage and support subordinates to use their strengths. As mentioned previously, support for strengths use is an important driving force of strengths use (Van Woerkom et al., 2016b). Thus, it is reasonable to predict that humble leadership has a positive impact on subordinates’ strengths use. In sum, we propose the following hypothesis:

**Hypothesis 2:** Humble leadership is positively related to strengths use.

#### Humble Leadership and Job Crafting

Job crafting as a specific form of proactive work behavior has been defined as the self-initiated changes that employees make in the task or in the relational boundaries of their work that are aimed at improving person–job fit (Wrzesniewski and Dutton, 2001; Tims et al., 2012). Job crafting is not explicitly authorized by the employer but initiated by employees (i.e., bottom–up) (Hornung et al., 2010), which is a critical potential path where organizations can gain competitive advantage (Esteves and Lopes, 2017). Wrzesniewski and Dutton (2001) put forward three forms of job crafting: task crafting, relational crafting, and cognitive crafting. Task crafting may be achieved by altering the type, number, content, or scope of tasks and work routines (Ghitulescu, 2006). Employees can craft relations with others by changing the range, nature, or the number of their interactions at work (Kooij et al., 2015). Cognitive crafting refers to the change employees make to views on work (Wrzesniewski and Dutton, 2001). Subsequently, Tims and Bakker (2010) provided another perspective of understanding job crafting using the job demands–resources theory as a framework and proposed four dimensions of job crafting: increasing structural job resources, social job resources, and challenge job demands as well as decreasing hindrance job demands (Hetland et al., 2018). Empirical studies have found that transformational leadership (Wang et al., 2017), paternalistic leadership (Tuan, 2018), willingness to change, impact of change (Petrou et al., 2015), and autonomy support (Slemp et al., 2015) could significantly predict job crafting of employees. From an integrative perspective, Niessen et al. (2016) investigated the needed abilities and reasons for job crafting and found that self-efficacy (can do), need for control, need for human connection, and need for positive self-image (reason to) are positively associated with job crafting.

Although researchers have shown a sophisticated understanding of the conception of job crafting and identified some important driving forces of job crafting, we still have not found explicit literature on the association of humble leadership with job crafting. In the current study, we thus attempt to address the gap and hypothesize that humble leadership has a positive correlation with job crafting. Humble leadership, conceptually, has significant interpersonal implications with a strong motive for learning from subordinates (Owens et al., 2013), which contributes to eliciting subordinates’ self-efficacy (Wang et al., 2018). Subordinates high in self-efficacy are more likely to believe that they can craft their job to achieve job objectives (Niessen et al., 2016). Therefore, humble leadership may have a positive correlation with job crafting. Moreover, humble leaders have an orientation toward subordinates and make the courageous decision to give up a certain portion of power so that subordinates have discretion in dimensions of their jobs (Chen et al., 2018). Such autonomy support encourages subordinates to craft their job (Slemp et al., 2015). Besides, qualitative research conducted by Owens and Hekman (2012) illustrated that leader-expressed humility has a positive association with employee engagement because humble leadership can cultivate the preconditions for employee engagement (Owens et al., 2013). A two-wave longitudinal study also demonstrated the work engagement of employees to be linked with job crafting (Lu et al., 2014). Accordingly, it is possible to expect humble leadership to relate to job crafting. Hence, we hypothesize the following:

**Hypothesis 3:** Human leadership is positively related to job crafting.

#### Strengths Use and Job Crafting

A substantial body of research has found that employees who utilize their strengths in the workplace are inclined to perform better and be more proactive (Cable et al., 2013; Dubreuil et al., 2014) and perform more helping behaviors and less counterproductive behaviors (Kong and Ho, 2016; Lavy and Littman-Ovadia, 2017; Littman-Ovadia et al., 2017). While strengths use can bring about numerous desirable outcomes, little is known about whether strengths use can positively affect job crafting. In the current study, we assume that strengths use has a positive association with job crafting.

When employees use their strengths at work, they can feel their most authentic self (Harzer and Ruch, 2013) and are more positive, energetic, and active (Bakker et al., 2019). Subsequently, they will engage more effort, time, and energy in work. That implicitly signifies that employees with higher levels of strengths use will experience higher work engagement. Indeed, a recent daily diary study also indicated that strengths use is positively related to work engagement (Bakker et al., 2019). As demonstrated earlier, work engagement is a significant and positive predictor of job crafting (Lu et al., 2014). Thus, we expect strengths used to positively relate to job crafting. In addition, in a weekly diary study, Van Woerkom et al. (2016b) pointed out that strengths use can predict a change in self-efficacy. Given that self-efficacy is a precursor of job crafting (Niessen et al., 2016), we also believe that the positive effects of strengths use can be conveyed to job crafting. Based on the above arguments, we postulate the following:

**Hypothesis 4:** Strengths use is positively related to job crafting.

#### The Mediating Role of Strengths Use

Strengths use was found to be not only linked with well-being and task performance (Littman-Ovadia and Steger, 2010; Wood et al., 2011; Kong and Ho, 2016) but also related to helping behaviors (Kong and Ho, 2016). In terms of helping behaviors, employees who use strengths at work can experience higher feelings of positive affect (Wood et al., 2011; Forest et al., 2012), and in turn, this leads to enhanced intention to help others (Kong and Ho, 2016); moreover, employees can gain more energy from strengths use (Dubreuil et al., 2014), which offers more resources that employees can deploy to help others (Kong and Ho, 2016). Given that helping behaviors is one aspect of OCB (Bachrach et al., 2007), we thus speculate that strengths use acts as the same role in enhancing OCB. Empirical evidence provides support for the notion. For example, Lavy and Littman-Ovadia (2017) concluded that strengths use can influence OCB by the broaden-and-build effect of positive emotions. Similar research also revealed that using signature strengths has a unique contribution to OCB (Littman-Ovadia et al., 2017). In addition, Van Woerkom and Meyers (2015) found that strengths-based psychological climate can significantly predict employees’ OCB, which also offers a piece of indirect evidence for the relationship between strengths use and OCB. Based on the previous prediction that humble leadership is positively related to strengths use and the above discussion, humble leadership may affect OCB by strengths use. Therefore, we posit the following:

**Hypothesis 5:** Strengths use mediates the relationship between humble leadership and OCB.

#### The Mediating Role of Job Crafting

In the current study, we assume that job crafting may play an important mediating role in the relationship between humble leadership and OCB. Job crafting as a form of proactive behaviors has been demonstrated to be associated with many valuable outcomes, such as colleague ratings of in-role performance (Bakker et al., 2012; Tims et al., 2015), work enjoyment (Tims et al., 2014), well-being (Tims et al., 2013), employees’ fit to the organization, job satisfaction (Kim et al., 2018), and intrinsic need satisfaction (Slemp and Vella-Brodrick, 2014). Bakker et al. (2012) illustrated that employees engaging in job crafting tend to mobilize their job resources and create a challenging work environment to foster enthusiasm and absorption. Also, when employees are dedicated to their work, they will be more likely to perform higher levels of OCB (Babcock-Roberson and Strickland, 2010). Accordingly, we believe that job crafting may have a positive association with OCB. A three-wave study indicated that when employees engage in job crafting, they can control their environment, which in turn results in higher self-efficacy and optimism; employees crafting their job can also experience a high level of hope by the process of goal setting and finding a way to achieve those objectives; more importantly, job crafting can help employees overcome some difficulties, which then leads to increased resiliency (Vogt et al., 2015). That implicitly means that job crafting has a significantly positive correlation with psychological capital. In line with the notion, Wingerden et al. (2016) also found a positive relationship between job crafting and psychological capital. Given the positive association of psychological capital with OCB (Norman et al., 2010), we believe that job crafting is positively related to OCB. Combining prior anticipation that humble leadership is positively related to job crafting with the above arguments, we can obtain the following hypothesis:

**Hypothesis 6:** Job crafting mediates the relationship between humble leadership and OCB.

#### The Sequential Mediation

Integrating all hypotheses mentioned above, we anticipate that the relationship between humble leadership and OCB can be mediated by strengths use and job crafting (a serial mediation). Prior research has shown strengths use to be associated with increased job crafting (Plomp et al., 2016). The main reason for the positive relationship between strengths use and job crafting is that when employees deploy their strengths at work, they are more likely to experience more energy and higher levels of authenticity (Mahomed and Rothmann, 2019), and these positive feelings provide crucial psychological conditions for job crafting (Kira et al., 2012; Lu et al., 2014). According to social learning theory, employees might learn some important behaviors by imitating the behaviors of role models such as leaders (Bavik et al., 2017). Employees who follow humble leaders have the same propensity to use strengths at work as leaders (Wang et al., 2018); such strengths use behavior contributes to higher levels of job crafting. As demonstrated earlier, job crafting has predictive value for OCB through the mechanism of psychological capital (Norman et al., 2010; Wingerden et al., 2016). Therefore, strengths use triggered by humble leadership will affect job crafting, which in turn leads to an improvement in OCB. Taken together, we formulate the following hypothesis:

**Hypothesis 7:** The relationship between humble leadership and OCB is mediated by strengths use and job crafting (sequential mediation).

**FIGURE 1:**
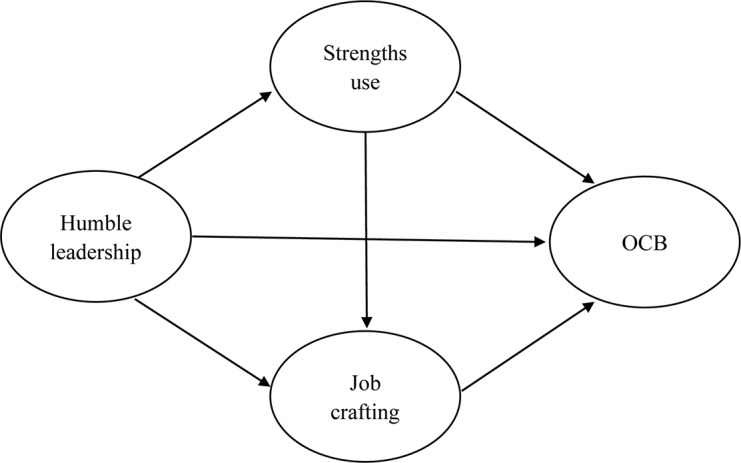
The proposed model.

The proposed model is presented in Figure 1.

## Materials and Methods

### Sample and Data Collection

A self-report research design was used in the current article. We adopted the convenience sampling to recruit participants. Participants were medical staff from a hospital in Beijing, China. An author of the present study contacted her friend working in the hospital and asked for her help to collect data. After obtaining consent from the manager and staff, we distributed electronic questionnaires to 316 medical staff in the hospital by WeChat (a type of communication tool) to gather data. Data were collected at two points in time spaced by a 2-week interval to reduce the common method variance (CMV). We set the electronic questionnaires as follows: only when the electronic questionnaire was completely filled can it be submitted successfully. In the first stage, participants completed a questionnaire consisting of demographic variables and scales of humble leadership, strengths use, and job crafting. After 2 weeks, participants completed a measure of OCB. Finally, 260 valid paired data were deployed to test our predictions, the valid response rate was 82.3%. Demographic characteristics were described in Table 1.

**TABLE 1:** Demographic characteristics of the participants (sample size, *N* = 260).

**Variables**	**Categories**	**Percentage (%)**
Gender	Male	0.8
	Female	99.2
Age	25 years old and under	16.5
	26–35 years	54.6
	36–45 years	16.9
	46 years and over	11.9
Tenure	5 years and below	33.5
	6–10 years	34.2
	11–15 years	11.5
	16 years and over	20.8
Education	Master’s degree	0.8
	Bachelor’s degree	61.2
	Associate and/or below qualification	38.0

### Measures

A five-point Likert scale (1 = strongly disagree, 2 = disagree, 3 = neutral, 4 = agree, 5 = strongly agree) was applied to rate all items of main variables (i.e., humble leadership, strengths use, job crafting, and OCB). Since four scales used in the current research were initially developed in English, they were translated into Chinese depending on the process of translation and back translation to ensure the equivalence of items (Brislin, 1970).

#### Humble Leadership

Humble leadership was measured with the nine-item scale developed by Owens et al. (2013), which was composed of three dimensions: willingness to see the self accurately, appreciation of others’ strengths and contributions, and teachability. One sample item was “My leader acknowledges when others have more knowledge and skills than him-or-herself.” The Cronbach α for this scale in the current study was 0.96, indicating excellent reliability.

#### Job Crafting

We assessed job crafting with the 15-item Job Crafting Questionnaire developed by Slemp and Vella-Brodrick (2013), which rates the degree to which employees engage in various forms of task, relational, and cognitive crafting (Slemp et al., 2015). One sample item was “Introduce new tasks you think suit your skills or interests.” The Cronbach α for this scale in the current study was 0.90, suggesting excellent reliability.

### Strengths Use

We evaluated strengths use using five items of Strengths Use and Deficit Correction (SUDCO) questionnaire developed by Van Woerkom et al. (2016a). One sample item was “In my job, I make the most of my strong points.” The Cronbach α for this scale in the current study was 0.83, indicating good reliability.

#### Organizational Citizenship Behavior

Organizational citizenship behavior was assessed with the 10-item scale used by Bachrach et al. (2007). The scale consists of two dimensions: helping (seven items) and civic virtue (three items). One sample item was “Help other employees out if someone falls behind in his/her work.” The Cronbach α of the scale in the current study was 0.92, demonstrating excellent reliability.

#### Control Variables

In line with previous studies, employees’ gender, age, and organizational tenure were selected as control variables (Lee and Allen, 2002; Cropanzano et al., 2003). Gender was coded: male = 1 and female = 2. Age was coded: 1 = no more than 25 years, 2 = 26–35 years, 3 = 36–45 years, 4 = 46–55 years, and 5 = 56 years and above. Organizational tenure was coded: 1 = no more than 5 years, 2 = 6–10 years, 3 = 11–15 years, 4 = 16 years and above.

## Results

### Descriptive Statistics

Table 2 reported means, standard deviations, and correlations of all study variables. The results showed that humble leadership was significantly and positively associated with strengths use (*r* = 0.38, *p* < 0.01), job crafting (*r* = 0.53, *p* < 0.01), and OCB (*r* = 0.30, *p* < 0.01), and strengths use was positively associated with job crafting (*r* = 0.62, *p* < 0.01) and OCB (*r* = 0.36, *p* < 0.01). Besides, the results also indicated that job crafting was positively associated with OCB (*r* = 0.49, *p* < 0.01).

**TABLE 2:** Means, standard deviations, and correlations.

**Variable**	***M***	***SD***	**1**	**2**	**3**	**4**	**5**	**6**
(1) Gender	1.99	0.09	−					
(2) Age	2.24	0.87	0.08	−				
(3) Tenure	2.20	1.12	0.06	0.84**	−			
(4) Humble leadership	3.94	0.75	−0.13*	−0.15*	−0.07	−		
(5) Strengths use	3.78	0.59	−0.08	0.03	0.06	0.38**	−	
(6) Job crafting	3.85	0.49	−0.16*	−0.13*	−0.07	0.53**	0.62**	−
(7) OCB	3.93	0.54	−0.07	−0.07	−0.05	0.30**	0.36**	0.49**

*p < 0.05; **p < 0.01.

### Discriminant Validity

To check the measurement model fit, we conducted a confirmatory factor analysis (CFA) in AMOS 21.0. Prior to performing CFA, in order to control for inflated measurement errors originating from multiple items for the latent variable and enhance the reliability and normality of the resulting measure (Nasser-Abu and Wisenbaker, 2006; Shi et al., 2015), three item parcels for humble leadership, three item parcels for job crafting, and two item parcels for OCB were created according to their dimensions. These item parcels were considered as indicators of corresponding constructs. Besides, all items of strengths use scale were viewed as indicators of strengths use. We selected six indices, including χ^2^/*df*, the root mean square error of approximation (RMSEA), comparative fit index (CFI), Tucker–Lewis index (TLI), incremental fit index (IFI), and goodness of fit index (GFI), to assess the overall model fit.

A four-factor model including humble leadership, strengths use, job crafting, and OCB was regarded as the baseline model. In order to examine the distinctiveness of the key constructs in the proposed model, we compared the baseline model with three alternative models. Table 3 reported the results of CFA. As shown in Table 3, the four-factor model exhibited adequate fit to the data: χ^2^ = 102.60, *df* = 59, χ^2^/*df* = 1.74, *p* < 0.001, RMSEA = 0.05, CFI = 0.98, TLI = 0.97, IFI = 0.98, GFI = 0.94. More importantly, there existed a significant difference in χ^2^ of the baseline model and three alternative models, which demonstrated that respondents could differentiate the four constructs very well. Therefore, the four constructs in the present study had a good discriminant validity.

**TABLE 3:** Results of CFAs: comparison of measurement models.

**Models**	**χ^2^**	***df***	**χ^2^/*df***	**RMSEA**	**CFI**	**TLI**	**IFI**	**GFI**	**SRMR**	**Δχ^2^ (Δ*df*)**
Baseline model	102.60	59	1.74	0.05	0.98	0.97	0.98	0.94	0.04	−
Three-factor model^a^	228.64	62	3.69	0.10	0.92	0.90	0.92	0.86	0.06	126.04*** (3)
Two-factor model^b^	767.25	64	11.99	0.21	0.65	0.57	0.65	0.64	0.11	664.65*** (5)
One factor model^c^	878.76	65	13.52	0.22	0.59	0.51	0.60	0.63	0.12	776.16*** (6)

^*a*^Strengths use and job crafting combined into one factor. ^*b*^Humble leadership, job crafting, and strengths use combined into one factor. ^*c*^All combined into one factor. ***p < 0.001.

#### Common Method Variance

Although the present study gathered the data at two points in time to control CMV, the self-report questionnaire survey might give rise to the CMV. Thus, we adopted the “controlling for the effects of a single unmeasured latent method factor” method to examine the degree of the CMV (Podsakoff et al., 2003). In accordance with prior studies (e.g., Ng et al., 2014; Xu and Lv, 2018), we established a new measurement model comprising a common method factor and four focal variables. All items were loaded on their theoretical constructs and the method factor. The results demonstrated that the new measurement model exhibited a good fit to the data (χ^2^ = 93.97, *df* = 58, χ^2^/*df* = 1.62, *p* < 0.01, RMSEA = 0.05, CFI = 0.98, TLI = 0.98, IFI = 0.98, GFI = 0.95). However, variance interpretation of the method factor was 12.75%, less than 25.00% (Williams et al., 1989). Hence, the CMV did not appear to be a serious threat to the interpretation of our results.

### Hypothesis Testing

According to Anderson and Gerbing’s (1988) suggestion, a two-step procedure was applied to examine our hypotheses. As shown in discriminant validity, the measurement model involving four latent variables (humble leadership, strengths use, job crafting, and OCB) reported an excellent fit to data. Moreover, all the factor loadings for the indicators of latent variables were significant at the 0.001 level, demonstrating that all the latent constructs can be represented by their corresponding indicators.

With respect to the structural model, three control variables were included in all structural models. The direct effect of humble leadership on OCB without mediators was first tested. The results suggested that the model (χ^2^ = 25.52, *df* = 18, χ^2^/*df* = 1.42, RMSEA = 0.04, CFI = 0.99, TLI = 0.99, IFI = 0.99, GFI = 0.98) showed an excellent fit to the data, and the directly standardized path (β = 0.33, *p* < 0.001) was significant, indicating that humble leadership has a positive effect on OCB. Hypothesis 1 was supported.

Second, we conducted SEM analysis for the proposed model. The results showed that the proposed model fits the data very well (χ^2^ = 148.04, *df* = 97, χ^2^/*df* = 1.53, RMSEA = 0.05, CFI = 0.98, TLI = 0.97, IFI = 0.98, GFI = 0.93, AIC = 226.04, ECVI = 0.87). However, the standardized path coefficients from humble leadership to OCB and from strengths use to OCB were not significant (humble leadership → OCB: β = −0.02, *p* > 0.05; strengths use → OCB: β = 0.03, *p* > 0.05). Thus, we modified the proposed model by deleting the two paths, and then the modified model was retested, which reported an excellent fit to the data (χ^2^ = 148.21, *df* = 99, χ^2^/*df* = 1.50, RMSEA = 0.04, CFI = 0.98, TLI = 0.97, IFI = 0.98, GFI = 0.93, AIC = 222.21, ECVI = 0.86). Although there was no significant difference between the modified model and the proposed model according to the fit indices, AIC value and the parsimony of the modified model were slightly smaller than those of the proposed model, suggesting that the modified model was more satisfactory.

**FIGURE 2:**
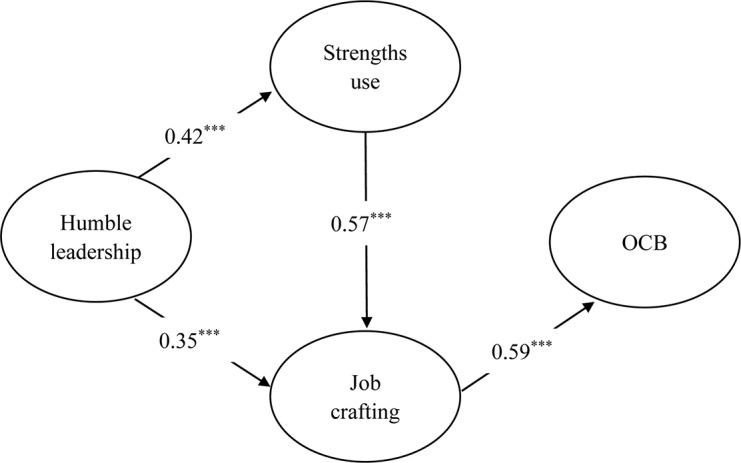
Results of SEM for the modified model.

The standardized path coefficients for the modified model were reported in Figure 2. The modified model explained 35.1% of variance in OCB. As shown in Figure 2, the standardized path coefficient between humble leadership and strengths use was 0.42 (*p* < 0.001), supporting Hypothesis 2; the standardized path coefficient between humble leadership and job crafting was 0.35 (*p* < 0.001), which is supportive of Hypothesis 3; the standardized path coefficient between strengths use and job crafting was 0.57 (*p* < 0.001), providing support for Hypothesis 4. As suggested previously, the standardized path coefficient from strengths use to OCB was not significant; thus, Hypothesis 5 was not supported.

We performed bootstrapping procedures (2,000 draws) with 95% bias-corrected confidence intervals to estimate the indirect effect in the revised model and its significance (MacKinnon et al., 2004). As reported in Table 4, the indirect effect of humble leadership on OCB via job crafting was significant, offering support for Hypothesis 6; the indirect effect of humble leadership on OCB through strengths use and job crafting (sequential mediation) was also significant, which was supportive of Hypothesis 7.

**TABLE 4:** Standardized indirect effects and 95% confidence intervals.

**Model pathways**	**Estimated**	**95% CI**	
		**Lower**	**Upper**
Humble leadership → Strengths use → Job crafting	0.15	0.09	0.22
Humble leadership → Job crafting → OCB	0.16	0.09	0.24
Strengths use → Job crafting → OCB	0.38	0.23	0.57
Humble leadership → Strengths use → Job crafting → OCB	0.11	0.06	0.18

Finally, a *post hoc* power analysis was carried out in GPower with a sample size of 260 and six predictor variables as baseline to examine the appropriateness and representativeness of our sample and findings. According to Cohen’s (1977) suggestion, the following effect sizes were used for this evaluation: small (*f*_2_ = 0.02), medium (*f*_2_ = 0.15), and large (*f*_2_ = 0.35). *Post hoc* power analysis showed that the power to detect obtained effects was 0.999 for the entire regression in the prediction of OCB at the 0.05 level beyond the value of 0.8 recommended by Cohen (1977) and Mustafa et al. (2016). As such, we can conclude that the power to detect small effects was enough with a sample of 260 and that our findings are appropriate and representative.

## Discussion

Researchers have identified many determinants of OCB (e.g., Lim et al., 2018; Khan et al., 2019). However, there is still a paucity of literature on the relationship between humble leadership and OCB. By a two-wave survey with a sample of 260 employees working in a hospital in Beijing, China, the present study examines the prediction that when subordinates experience higher levels of humble leadership, they will respond to this with higher OCB. Moreover, this paper tests the mediational roles of strengths use and job crafting, respectively, in the relationship between humble leadership and OCB. More importantly, this paper further examines the sequential mediation effect of strengths use and job crafting on the relationship between humble leadership and OCB. The findings of this paper showed that humble leadership is positively related to OCB, strengths use, and job crafting and that job crafting plays a significant and positive mediating role in the relationship between humble leadership and OCB. More importantly, strengths use and job crafting serve a sequential mediation role in the relationship between humble leadership and OCB. However, the mediating role of strengths use in the humble leadership–OCB relationship is not significant. These findings have several theoretical and practical implications, which are discussed below.

### Theoretical Implications

The present study makes two main theoretical contributions to OCB literature. First, by investigating the relationship between humble leadership and OCB, our study enriches the literature on antecedents of OCB. Crant (2000) pointed out that leaders serve as a crucial role in facilitating employee proactive behavior (e.g., OCB). Although scholars appreciated the importance of leadership to OCB and found that transformational leadership (Jiao et al., 2011), attributional charismatic leadership (Deluga, 1995), shared leadership (Khasawneh, 2011), and developmental leadership (Yan and Chen, 2013) are positively related to OCB, less is known about the association of humble leadership with OCB. In line with previous research that found humble leadership to positively relate to employee proactive behavior (Chen et al., 2018), our study indicated that humble leadership is positively related to OCB, which adds to the literature on antecedents of OCB.

Second, the current study stresses the importance of the mediating role of job crafting, which contributes to unpacking the “black box” in the humble leadership–OCB relationship. Our study showed that job crafting acts as a mediator in the relationship between humble leadership and OCB. Concretely, when subordinates perceive a high level of humble leadership, they will have the feelings of being empowered by leaders and having more autonomy to make decisions at work (Chen et al., 2018); they are more likely to engage in work (Wang et al., 2018) and experience higher self-efficacy (Owens et al., 2013). Such positive outcomes induced by humble leadership will lead to increased job crafting. However, our study did not provide evidence for the mediating effect of strengths use on the humble leadership–OCB relationship. According to the results of data analysis, a possible reason for this finding is that for employees working in a hospital, strengths use does not directly but indirectly affect employees’ OCB; that is, the effect of strengths use on OCB should be understood through the mediating mechanism of job crafting. Previous research conducted by Van Woerkom and Meyers (2015) suggested that positive affect fully mediated the relationship between strengths-based psychological climate and OCB, which to a certain extent offers support for the argument. In a word, this study can help us to understand the underlying process mechanism behind the humble leadership–OCB relationship.

Further study found that strengths use and job crafting served as a tandem mediating effect on the relationship between humble leadership and OCB, which contributes to our understanding of deeply potential mechanisms behind the humble leadership–OCB relationship. Prior research revealed that psychological capital (Wang et al., 2018) and psychological empowerment (Chen et al., 2018) respectively played important mediational roles in the effects of humble leadership. However, to the best of our knowledge, no prior research explored the underlying process mechanisms of effects of humble leadership from the perspective of strengths use and job crafting. Our study addressed this concern and found that humble leadership could affect subordinates’ strengths use and then lead to increased job crafting, ultimately promoting OCB of subordinates. The result unravels a more complex process mechanism underlying the relationship between humble leadership and OCB, which contributes to deepening our understanding of the humble leadership–OCB relationship.

### Practical Implications

The current study has some important practical implications for organizations. First, this study found that humble leadership is effective in promoting employees’ OCB. Thus, to enhance employees’ OCB, organizations should promote employees with a high level of humble characteristics to be a leader or enable present leaders to learn more knowledge on humble leadership by training projects. Second, as the current study indicated that humble leadership has an indirect impact on OCB through the mediator, namely, job crafting, organizations should create conditions under which employees’ job crafting can be elevated. For example, organizations can provide employees with more autonomy to create the condition eliciting job crafting (Slemp et al., 2015). Third, the current study demonstrated that strengths use and job crafting play a significantly sequential mediating role in the relationship between humble leadership and OCB, which signifies that promoting employees’ strengths use is quite important for enhancing employees’ OCB. Thus, we propose that organizations should help employees to identify and develop their strengths to encourage them to leverage their strengths at work. In addition, providing employees with higher autonomy support is also an important way of improving employees’ strengths use (Kong and Ho, 2016).

### Limitations and Directions for Future Research

The current study is not without limitations. First, although this study adopted a two-wave survey separated by a 2-week interval to collect data, we still cannot make robust causal inferences about the relationship between humble leadership and strengths use, job crafting, and OCB due to the shorter time interval in the process of data collecting. Hence, future research should conduct quasi-experiment or longitudinal research to further examine the causal relationship among humble leadership, strengths use, job crafting, and OCB. Second, in the current study, almost all of participants are female from a hospital, which may raise the concern about the generalizability of our findings in that males and females might have a significant difference in cognition in leadership (Hyde, 1981). So it is quite necessary for researchers to conduct research on the relationships between humble leadership, strengths use, job crafting, and OCB using a more gender-balanced sample to expand the generalizability of our findings. Finally, the current study merely investigated the complex process mechanisms underlying the relationship between humble leadership and OCB. However, we still have little knowledge on the boundary conditions of the humble leadership–OCB relationship. Therefore, future research should try to identify the boundary conditions of the relationship between humble leadership and OCB.

## Data Availability Statement

The datasets analyzed in this article are not publicly available. Requests to access the datasets should be directed to believedh@126.com.

## Ethics Statement

The present study was carried out in accordance with the recommendations of the Ethics Committee of the North China Electric Power University with written informed consent from all subjects. All the participants were asked to read and approve this ethical consent before taking part in the present study and followed it in the process of research. The protocol was approved by the Ethics Committee of the North China Electric Power University.

## Author Contributions

HD designed the research and wrote the manuscript. XC collected the research data. EY and YL analyzed the data, and they, together with KA, amended the manuscript.

## Conflict of Interest

The authors declare that the research was conducted in the absence of any commercial or financial relationships that could be construed as a potential conflict of interest.

## References

[B1] Ahmad I. Zafar M. A. (2018). Impact of psychological contract fulfillment on organizational citizenship behavior. *Int. J. Contemp. Hosp. Manag.* 30 1001–1015. 10.1108/IJCHM-12-2016-0659

[B2] Anderson J. Gerbing D. W. (1988). Structural equation modeling in practice: a review and recommended two-step approach. *Psychol. Bull.* 103 411–423. 10.1037//0033-2909.103.3.411

[B3] Babcock-Roberson M. E. Strickland O. J. (2010). The relationship between charismatic leadership, work engagement, and organizational citizenship behaviors. *J. Psychol.* 144 313–326. 10.1080/00223981003648336 20461933

[B4] Bachrach D. G. Wang H. Bendoly E. Zhang S. (2007). Importance of organizational citizenship behavior for overall performance evaluation: comparing the role of task interdependence in China and the USA. *Manag. Organ. Rev.* 3 255–276. 10.1111/j.1740-8784.2007.00071.x

[B5] Bakker A. B. Hetland J. Kjellevold-Olsen O. Espevik R. (2019). Daily strengths use and employee well-being: the moderating role of personality. *J. Occup. Organ. Psychol.* 92 144–168. 10.1111/joop.12243

[B6] Bakker A. B. Tims M. Derks D. (2012). Proactive personality and job performance: the role of job crafting and work engagement. *Hum. Relat.* 65 1359–1378. 10.1177/0018726712453471

[B7] Bakker A. B. van Woerkom M. (2018). Strengths use in organizations: a positive approach of occupational health. *Can. Psychol.* 59 38–46. 10.1037/cap0000120

[B8] Basford T. E. Offermann L. R. Behrend T. S. (2014). Please accept my sincerest apologies: examining follower reactions to leader apology. *J. Bus. Ethics* 119 99–117. 10.1007/s10551-012-1613-y

[B9] Bavik A. Bavik Y. L. Tang P. M. (2017). Servant leadership, employee job crafting, and citizenship behaviors: a cross-level investigation. *Corn. Hosp. Q.* 58 364–373. 10.1177/1938965517719282

[B10] Becton J. B. Carr J. C. Mossholder K. W. Walker H. J. (2017). Differential effects of task performance, organizational citizenship behavior, and job complexity on voluntary turnover. *J. Bus. Psychol.* 32 495–508. 10.1007/s10869-016-9461-x

[B11] Botha C. Mostert K. (2014). A structural model of job resources, organizational and individualstrengths use and work engagement. *SA J. Indust. Psychol.* 40 1–11. 10.4102/sajip.v40i1.1135

[B12] Brislin R. W. (1970). Back-translation for cross-cultural research. *J. Cross Cult. Psychol.* 1 185–216. 10.1177/135910457000100301

[B13] Buckingham M. Clifton D. O. (2001). *Now, Discover Your Strengths.* New York, NY: Free Press.

[B14] Cable D. M. Gino F. Staats B. R. (2013). Breaking them in or eliciting their best? reframing socializationaround newcomers’ authentic self-expression. *Admin. Sci. Q.* 58 1–36. 10.1177/0001839213477098

[B15] Chen Y. Liu B. Zhang L. Qian S. (2018). Can leader “humility” spark employee “proactivity”? The mediating role of psychological empowerment. *Leadersh. Organ. Dev. J.* 39 326–339. 10.1108/LODJ-10-2017-0307 30558271

[B16] Cohen J. (1977). *Statistical Power Analysis for the Behavioral Sciences.* New York, NY: Academic Press.

[B17] Crant J. M. (2000). Proactive behavior in organizations. *J. Manag.* 26 435–462. 10.1016/S0149-2063(00)00044-1

[B18] Cropanzano R. Rupp D. E. Byrne Z. S. (2003). The relationship of emotional exhaustion to work attitudes, job performance, and organizational citizenship behaviors. *J. Appl. Psychol.* 88 160–169. 10.1037/0021-9010.88.1.160 12675403

[B19] Dai Y. D. Hou Y. H. Chen K. Y. Zhuang W. L. (2018). To help or not to help: antecedents of hotel employees’ organizational citizenship behavior. *Inter. J. Contemp. Hosp. Manag.* 30 1293–1313. 10.1108/IJCHM-03-2016-0160

[B20] Deluga R. J. (1995). The relationship between attributional charismatic leadership and organizational citizenship behavior. *J. Appl. Soc. Psychol.* 25 1652–1669. 10.1111/j.1559-1816.1995.tb02638.x

[B21] Dubreuil P. Forest J. Courcy F. (2014). From strengths use to work performance: the role of harmonious passion, subjective vitality, and concentration. *J. Posit. Psychol.* 9 335–349. 10.1080/17439760.2014.898318

[B22] Dussault M. (2006). Teachers’ self-efficacy and organizational citizenship behaviors. *Psychol. Rep.* 98 427–432. 10.2466/PR0.98.2.427-432 16796098

[B23] Esteves T. Lopes M. P. (2017). Leading to crafting: the relation between leadership perception and nurses job crafting. *West. J. Nurs. Res.* 39 763–783. 10.1177/0193945916659507 27432346

[B24] Forest J. Mageau G. A. Crevier-Braud L. Bergeron E. Dubreuil P. Lavigne G. L. (2012). Harmonious passion as an explanation of the relation between signature strengths’ use and well-being at work: test of an intervention program. *Hum. Relat.* 65 1233–1252. 10.1177/0018726711433134

[B25] Ghitulescu B. E. (2006). *Shaping Tasks and Relationships at Work: Examining the Antecedents and Consequences of Employee Job Crafting.* Pittsburgh, PA: University of Pittsburgh.

[B26] Govindji R. Linley P. A. (2007). Strengths use, self-concordance and well-being: implications for strengths coaching and coaching psychologists. *Int. Coach. Psychol. Rev.* 2 143–153.

[B27] Harzer C. Ruch W. (2013). The application of signature character strengths and positive experiences atwork. *J. Happ. Stud.* 14 965–983. 10.1007/s10902-012-9364-0

[B28] Hetland J. Hetland H. Bakker A. Demerouti E. (2018). Daily transformational leadership and employee job crafting: the role of promotion focus. *Eur. Manag. J.* 36 746–756. 10.1016/j.emj.2018.01.002

[B29] Hornung S. Rousseau D. M. Glaser J. Angerer P. Weigl M. (2010). Beyond top-down and bottom-up work redesign: customizing job content through idiosyncratic deals. *J. Organ. Behav.* 31 187–215. 10.1002/job.625

[B30] Hyde J. S. (1981). How large are cognitive gender differences? A meta-analysis using w^2^ and d. *Am. Psychol.* 36 892–901. 10.1037/0003-066X.36.8.892

[B31] Jiao C. Richards D. A. Zhang K. (2011). Leadership and organizational citizenship behavior: OCB-specific meanings as mediators. *J. Bus. Psychol.* 26 11–25. 10.1007/s10869-010-9168-3

[B32] Kao R. H. (2017). Task-oriented work characteristics, self-efficacy, and service-oriented organizational citizenship behavior. *Pers. Rev.* 46 718–739. 10.1108/PR-08-2015-0234

[B33] Katz D. (1964). The motivational basis of organizational behavior. *Syst. Res. Behav. Sci.* 9 131–146. 10.1002/bs.3830090206 5888769

[B34] Khan N. A. Khan A. N. Gul S. (2019). Relationship between perception of organizational politics and organizational citizenship behavior: testing a moderated mediation model. *Asian Bus. Manag.* 18 122–141. 10.1057/s41291-018-00057-9

[B35] Khasawneh S. (2011). Shared leadership and organizational citizenship behaviour in jordanian public universities: developing a global workforce for the 21st century. *Educ. Manag. Admin. Leadersh.* 39 621–634. 10.1177/1741143211408447

[B36] Kim H. Im J. Qu H. Namkoong J. (2018). Antecedent and consequences of job crafting: an organizational level approach. *Int. J. Contemp. Hosp. Manag.* 30 1863–1881. 10.1108/IJCHM-01-2017-0040

[B37] Kira M. Balkin D. B. San E. (2012). Authentic work and organizational change: longitudinal evidence from a merger. *J. Chang. Manag.* 12 31–51. 10.1080/14697017.2011.652374

[B38] Kong D. T. Ho V. T. (2016). A self-determination perspective of strengths use at work: examining its determinant and performance implications. *J. Posit. Psychol.* 11 15–25. 10.1080/17439760.2015.1004555

[B39] Kooij D. T. A. M. Tims M. Kanfer R. (2015). “Successful aging at work: the role of job crafting,” in *Aging Workers and the Employee-Employer Relationship*, eds Bal P. Kooij D. Rousseau D., (Cham: Springer).

[B40] Lavy S. Littman-Ovadia H. (2017). My better self: using strengths at work and work productivity, organizational citizenship behavior, and satisfaction. *J. Career Dev.* 44 95–109. 10.1177/0894845316634056

[B41] Lee K. Allen N. J. (2002). Organizational citizenship behavior and workplace deviance: the role of affect and cognitions. *J. Appl. Psychol.* 87 131–142. 10.1037/0021-9010.87.1.131 11916207

[B42] Lim S. H. Han S. S. Joo Y. S. (2018). Effects of nurses’ emotional intelligence on their organizational citizenship behavior, with mediating effects of leader trust and value congruence. *Jap. J. Nurs. Sci.* 25 12–17. 10.1111/jjns.12206 29464858

[B43] Littman-Ovadia H. Lavy S. Boiman-Meshita M. (2017). When theory and research collide: examiningcorrelates of signature strengths use at work. *J. Happ. Stud.* 18 527–548. 10.1007/s10902-016-9739-8

[B44] Littman-Ovadia H. Steger M. (2010). Character strengths and well-being among volunteers and employees: toward an integrative model. *J. Posit. Psychol.* 5 419–430. 10.1080/17439760.2010.516765

[B45] Liu W. X. Mao J. H. Chen X. (2017). Leader humility and team innovation: investigating the substituting role of task interdependence and the mediating role of team voice climate. *Front. Psychol.* 8:1115. 10.3389/fpsyg.2017.01115 28713316 PMC5492832

[B46] Lu C. Q. Wang H. J. Lu J. J. Du D. Y. Bakker A. B. (2014). Does work engagement increase person–job fit? the role of job crafting and job insecurity. *J. Vocat. Behav.* 84 142–152. 10.1016/j.jvb.2013.12.004

[B47] MacKinnon D. P. Lockwood C. M. Williams J. (2004). Confidence limits for the indirect effect: distribution of the product and resampling methods. *Multivar. Behav. Res.* 39 99–128. 10.1207/s15327906mbr3901_4 20157642 PMC2821115

[B48] Mahomed F. E. Rothmann S. (2019, in press). Strength use, training and development, thriving, and intention to leave: the mediating effects of psychological need satisfaction. *South Afr. J. Psychol.* 1–15. 10.1177/0081246319849030

[B49] Mostafa A. M. S. (2018). Ethical leadership and organizational citizenship behaviours: the moderating role of organizational identification. *Eur. J. Work Organ. Psychol.* 27 1–9. 10.1080/1359432X.2018.1470088 24684078

[B50] Mustafa M. Martin L. Hughes M. (2016). Psychological ownership, job satisfaction, and middle manager entrepreneurial behavior. *J. Leadersh. Organ. Stud.* 23 272–287. 10.1177/1548051815627360

[B51] Nasra M. A. Heilbrunn S. (2016). Transformational leadership and organizational citizenship behavior in the Arab educational system in Israel: the impact of trust and job satisfaction. *Educ. Manag. Admin. Leadersh.* 44 380–396. 10.1177/1741143214549975

[B52] Nasser-Abu A. F. Wisenbaker J. (2006). A monte carlo study investigating the impact of item parceling strategies on parameter estimates and their standard errors in CFA. *Struct. Equat. Model.* 13 204–228. 10.1207/s15328007sem1302_3

[B53] Newman A. Schwarz G. Cooper B. Sendjaya S. (2017). How servant leadership influences organizational citizenship behavior: the roles of LMX, empowerment, and proactive personality. *J. Bus. Ethics* 145 49–62. 10.1007/s10551-015-2827-6

[B54] Ng T. W. H. Feldman D. C. Butts M. M. (2014). Psychological contract breaches and employee voice behaviour: the moderating effects of changes in social relationships. *Eur. J. Work Organ. Psychol.* 23 537–553. 10.1080/1359432X.2013.766394

[B55] Niessen C. Weseler D. Kostova P. (2016). When and why do individuals craft their jobs? the role of individual motivation and work characteristics for job crafting. *Hum. Relat.* 69 1287–1313. 10.1177/0018726715610642

[B56] Norman S. M. Avey J. B. Nimnicht J. L. Graber Pigeon N. (2010). The interactive effects of psychological capital and organizational identity on employee organizational citizenship and deviance behaviors. *J. Leadersh. Organ. Stud.* 17 380–391. 10.1177/1548051809353764

[B57] Organ D. W. (1988). *Organizational Citizenship Behavior: The GoodSoldier Syndrome.* Lexington, MA: Lexington Books.

[B58] Ou A. Y. Seo J. Choi D. Hom P. W. (2017). When can humble top executives retain middle managers? The moderating role of top management team fault lines. *Acad. Manag. J.* 60 1915–1931. 10.5465/amj.2015.1072

[B59] Owens B. P. Hekman D. R. (2012). Modeling how to grow: an inductive examination of humble leader behaviors, contingencies, and outcomes. *Acad. Manag. J.* 55 787–818. 10.5465/amj.2010.0441

[B60] Owens B. P. Hekman D. R. (2016). How does leader humility influence team performance? exploring the mechanisms of contagion and collective promotion focus. *Acad. Manag. J.* 59 1088–1111. 10.5465/amj.2013.0660

[B61] Owens B. P. Johnson M. D. Mitchell T. R. (2013). Expressed humility in organizations: implications for performance, teams, and leadership. *Organ. Sci.* 24 1517–1538. 10.1287/orsc.1120.0795

[B62] Owens B. P. Wallace A. S. Waldman D. A. (2015). Leader narcissism and follower outcomes: the counterbalancing effect of leader humility. *J. Appl. Psychol.* 100 1203–1213. 10.1037/a0038698 25621592

[B63] Petrou P. Demerouti E. Schaufeli W. B. (2015). Job crafting in changing organizations: antecedents and implications for exhaustion and performance. *J. Occup. Health Psychol.* 20 470–480. 10.1037/a0039003 25798717

[B64] Plomp J. Tims M. Akkermans J. Khapova S. N. Jansen P. G. Bakker A. B. (2016). Career competencies and job crafting: how proactive employees influence their well-being. *Career Dev. Int.* 21 587–602. 10.1108/CDI-08-2016-0145

[B65] Podsakoff N. P. Whiting S. W. Podsakoff P. M. Blume B. D. (2009). Individual- and organizational-level consequences of organizational citizenship behaviors: a meta-analysis. *J. Appl. Psychol.* 94 122–141. 10.1037/a0013079 19186900

[B66] Podsakoff P. M. Mackenzie S. B. (1997). Impact of organizational citizenship behavior on organizational performance: a review and suggestion for future research. *Hum. Perform.* 10 133–151. 10.1207/s15327043hup1002-5

[B67] Podsakoff P. M. Mackenzie S. B. Lee J. Y. Podsakoff N. P. (2003). Common method biases in behavioral research: a critical review of the literature and recommended remedies. *J. Appl. Psychol.* 88 879–903. 10.1037/0021-9010.88.5.879 14516251

[B68] Podsakoff P. M. MacKenzie S. B. Moorman R. H. Fetter R. (1990). Transformational leader behaviors and their effects on followers’ trust in leader, satisfaction, and organizational citizenship behaviors. *Leadersh. Q.* 1 107–142. 10.1016/1048-9843(90)90009-7

[B69] Rego A. Owens B. Kai C. Y. Bluhm D. Cunha M. P. E. Silard T. (2017a). Leader humility and team performance: exploring the mediating mechanisms of team psychological capital and task allocation effectiveness. *J. Manag.* 20 1–25. 10.1177/0149206316688941

[B70] Rego A. Owens B. Leal S. Melo A. I. Cunha M. P. E. Gonçalves L. (2017b). How leader humility helps teams to be humbler, psychologically stronger, and more effective: a moderated mediation model. *Leadersh. Q.* 28 639–658. 10.1016/j.leaqua.2017.02.002

[B71] Schlechter A. F. Maharaj I. (2007). Meaning in life and meaning of work: relationships with organizational citizenship behaviour, commitment and job satisfaction. *Manag. Dyn.* 26 24–41.

[B72] Shi M. Yan X. You X. Li J. (2015). Core self-evaluations, emotional intelligence and job satisfaction in Chinese soldiers. *Soc. Indic. Res.* 124 221–229. 10.1007/s11205-014-0784-6

[B73] Slemp G. R. Kern M. L. Vella-Brodrick D. A. (2015). Workplace well-being: the role of job crafting and autonomy support. *Psychol. Well Being* 1 1–17. 10.1186/s13612-015-0034-y

[B74] Slemp G. R. Vella-Brodrick D. A. (2013). The Job Crafting Questionnaire: a new scale to measure the extent to which employees engage in job crafting. *Int. J. Wellbeing* 3 126–146. 10.5502/ijw.v3i2.1

[B75] Slemp G. R. Vella-Brodrick D. A. (2014). Optimising employee mental health: the relationship between intrinsic need satisfaction, job crafting, and employee well-being. *J. Happ. Stud.* 15 957–977. 10.1007/s10902-013-9458-3

[B76] Smith C. A. Organ D. W. Near J. P. (1983). Organizational citizenship behavior: its nature and antecedents. *J. Appl. Psychol.* 68 653–663. 10.1037/0021-9010.68.4.653

[B77] Tims M. Bakker A. Derks D. (2014). Daily job crafting and the self-efficacy – performance relationship. *J. Manag. Psychol.* 29 490–507. 10.1108/JMP-05-2012-0148

[B78] Tims M. Bakker A. B. (2010). Job crafting: towards a new model of individual jobredesign. *South Afr. J. Indust. Psychol.* 36 1–9. 10.4102/sajip.v36i2.841

[B79] Tims M. Bakker A. B. Derks D. (2012). Development and validation of the job crafting scale. *J. Vocat. Behav.* 80 173–186. 10.1016/j.jvb.2011.05.009

[B80] Tims M. Bakker A. B. Derks D. (2013). The impact of job crafting on job demands, job resources, and well-being. *J. Occup. Health Psychol.* 18 230–240. 10.1037/a0032141 23506549

[B81] Tims M. Bakker A. B. Derks D. (2015). Job crafting and job performance: a longitudinal study. *Eur. J. Work Organ. Psychol.* 24 914–928. 10.1080/1359432X.2014.969245

[B82] Tuan L. T. (2018). Behind the influence of job crafting on citizen value co-creation with the public organization: joint effects of paternalistic leadership and public service motivation. *Public Manag. Rev.* 20 1–29. 10.1080/14719037.2018.1430247

[B83] Van Woerkom M. Meyers M. C. (2015). My strengths count! effects of a strengths-based psychological climate on positive affect and job performance. *Hum. Resour. Manag.* 54 81–103. 10.1002/hrm.21623

[B84] Van Woerkom M. Mostert K. Els C. Bakker A. B. de Beer L. Rothmann S. Jr. (2016a). Strengths use and deficit correction in organizations: development and validation of a questionnaire. *Eur. J. Work Organ. Psychol.* 25 960–975. 10.1080/1359432X.2016.1193010

[B85] Van Woerkom M. Oerlemans W. Bakker A. B. (2016b). Strengths use and work engagement: a weekly diary study. *Eur. J. Work Organ. Psychol.* 25 384–397. 10.1080/1359432X.2015.1089862

[B86] Vogt K. Hakanen J. J. Brauchli R. Jenny G. J. Bauer G. F. (2015). The consequences of job crafting: a three-wave study. *Eur. J. Work Organ. Psychol.* 25 1–10.1.

[B87] Walumbwa F. O. Hartnell C. A. Oke A. (2010). Servant leadership, procedural justice climate, service climate, employee attitudes, and organizational citizenship behavior: a cross-level investigation. *J. Appl. Psychol.* 95 517–529. 10.1037/a0018867 20476830

[B88] Wang H. J. Demerouti E. Le Blanc P. (2017). Transformational leadership, adaptability, and job crafting: the moderating role of organizational identification. *J. Vocat. Behav.* 100 185–195. 10.1016/j.jvb.2017.03.009

[B89] Wang Y. Liu J. Zhu Y. (2018). How does humble leadership promote follower creativity? the roles of psychological capital and growth need strength. *Leadersh. Organ. Dev. J.* 39 507–521. 10.1108/LODJ-03-2017-0069

[B90] Williams L. J. Cote J. A. Buckley M. R. (1989). Lack of method variance in self-reported affect and perceptions at work: reality or artifact? *J. Appl. Psychol.* 74 462–468. 10.1037/0021-9010.74.3.462

[B91] Wingerden J. V. Bakker A. B. Derks D. (2016). A test of a job demands-resources intervention. *J. Manag. Psychol.* 31 686–701. 10.1108/JMP-03-2014-0086

[B92] Wood A. M. Linley P. A. Maltby J. Kashdan T. B. Hurling R. (2011). Using personal and psychological strengths leads to increases in well-being over time: a longitudinal study and the development of the strengths use questionnaire. *Pers. Individ. Differ.* 50 15–19. 10.1016/j.paid.2010.08.004

[B93] Wrzesniewski A. Dutton J. E. (2001). Crafting a job: revisioning employees as active crafters of their work. *Acad. Manag. Rev.* 26 179–201. 10.5465/amr.2001.4378011

[B94] Xu T. Lv Z. (2018). HPWS and unethical pro-organizational behavior: a moderated mediation model. *J. Manag. Psychol.* 33 265–278. 10.1108/JMP-12-2017-0457

[B95] Yan Z. Chen C. C. (2013). Developmental leadership and organizational citizenship behavior: mediating effects of self-determination, supervisor identification, and organizational identification. *Leadersh. Q.* 24 534–543. 10.1016/j.leaqua.2013.03.007

[B96] Yang Q. Wei H. (2017). The impact of ethical leadership on organizational citizenship behavior: the moderating role of workplace ostracism. *Leadersh. Organ. Dev. J.* 39 100–113. 10.1108/LODJ-12-2016-0313

